# MK591 (Quiflapon), a 5-lipoxygenase inhibitor, kills pancreatic cancer cells via downregulation of protein kinase C-epsilon

**DOI:** 10.3389/fonc.2024.1387535

**Published:** 2024-04-30

**Authors:** Jitender Monga, Ritisha Ghosh, Rohith Guddeti, Dhananjay Chitale, Gazala Khan, Jagadananda Ghosh

**Affiliations:** ^1^ Department of Urology, Henry Ford Health System, Detroit, MI, United States; ^2^ Department of Pathology, Henry Ford Health System, Detroit, MI, United States; ^3^ Henry Ford Cancer Institute, Henry Ford Health System, Detroit, MI, United States

**Keywords:** MK591, 5-lipoxygenase, pancreatic cancer, apoptosis, PKC-epsilon, gemcitabine

## Abstract

**Introduction:**

Pancreatic tumors and cell lines derived from them exhibit elevated expression of 5-lipoxygenase (5-Lox), whereas non-tumor glands or normal cells do not exhibit this overexpression. Arachidonic acid stimulates pancreatic cancer cell growth via metabolic conversion through the 5-Lox pathway, and inhibition of 5-Lox activity decreases the viability of pancreatic cancer cells. However, the downstream signaling mechanisms through which 5-Lox exerts its effects on the survival of pancreatic cancer cells remain to be elucidated.

**Methods:**

The effects of 5-Lox inhibition on cell proliferation, apoptosis, and invasive potential were investigated in pancreatic cancer cells. The protein expression was analyzed by Western blot. Apoptosis was analyzed by Annexin-V binding assay and by detecting the degradation of chromatin-DNA to nucleosomal fragments. The protein kinase C-epsilon (PKCε) activity was measured by an immunoprecipitation-kinase assay. The *in vivo* effects of MK591 were evaluated in pancreatic tumor xenograft model.

**Results:**

MK591, a specific inhibitor of 5-Lox activity, killed pancreatic cancer cells via induction of apoptosis, involving externalization of phosphatidylserine, cleavage of PARP (poly-ADP ribose polymerase) and degradation of chromatin DNA to nucleosomes. MK591 effectively blocked *in vitro* invasion and soft-agar colony formation by pancreatic cancer cells and decreased pancreatic tumor growth in nude mice xenografts. Furthermore, inhibition of 5-Lox downregulated K-Ras and inhibited phosphorylation of c-Raf and ERKs. Interestingly, 5-Lox inhibition induced apoptosis in pancreatic cancer cells without the inhibition of Akt but the protein level of PKCε was dramatically downregulated. Furthermore, inhibition of 5-Lox decreased the phosphorylation of Stat3 at Serine-727. Pre-treatment of pancreatic cancer cells with peptide activators of PKCε prevented apoptosis induced by 5-Lox inhibition, suggesting that the mechanism by which 5-Lox inhibition causes cell death in pancreatic cancer involves downregulation of PKCε. The combination of low doses of MK591 and gemcitabine synergistically reduced the oncogenic phenotype and killed pancreatic cancer cells by inducing apoptosis.

**Discussion:**

These findings indicate that inhibition of 5-Lox interrupts an Akt-independent, PKCε-dependent survival mechanism in pancreatic cancer cells and suggest that metabolism of arachidonic acid through the 5-Lox pathway plays an integral part in the survival of pancreatic cancer cells via signaling through PKCε, an oncogenic, pro-survival serine/threonine kinase.

## 1 Introduction

Pancreatic cancer is an aggressive, lethal form of malignancy and currently it is the fourth leading cause of cancer-related deaths in the United States (1–4). Though significant progress has been made in understanding the pathobiology of pancreatic cancer, most patients continue to present with advanced metastatic disease, and do not respond to targeted therapies. The genetic landscape of pancreatic cancer shows near-ubiquitous activating mutations of the K-Ras oncogene (5–9). However, attempts to develop agents to target the K-Ras oncogenic signaling and specifically kill pancreatic cancer cells have yielded limited benefits (10–13). Gemcitabine, with or without nab-paclitaxel, or with cisplatin, is a common line of therapy for pancreatic cancer, but while most patients initially respond to gemcitabine therapy, advanced disease invariably develops which eventually metastasize to distant organs and becomes lethal (14–18). Thus, management of pancreatic cancer remains a major problem primarily because of the failures of currently available therapies to prevent the morbidity and mortality associated with gemcitabine-resistant pancreatic cancer. Emerging principles of effective therapy indicates that anti-cancer agents should effectively kill and eliminate cancer cells from the system without affecting the non-cancer cells. However, lack of proper knowledge about critical molecular mechanisms in pancreatic cancer cells is hindering development of effective regimen against pancreatic cancer. Thus, identification and characterization of novel mechanisms which play specific and critical roles in pancreatic cancer cell survival are of utmost significance for development of effective therapeutic strategies to control this deadly disease.

Large scale epidemiological studies as well as experiments with laboratory animals suggested that there exists a link between consumption of high-fat diets and occurrence of clinically evident pancreatic cancer (19, 20), indicating that dietary fatty acids and metabolites may play an important role in the promotion and progression phases of pancreatic cancer via enhanced growth and survival characteristics of pancreatic cancer cells. An omega-6, polyunsaturated fatty acid, arachidonic acid, stimulates pancreatic cancer cell growth via metabolic conversion through the 5-Lox pathway, and inhibition of 5-Lox activity decreases the viability of pancreatic cancer cells (21–24). However, downstream mechanisms which mediate the survival-promoting effects of 5-Lox activity in pancreatic cancer cells have yet to be characterized. We found that in some cancer cells (e.g., prostate cancer cells) 5-Lox is continuously active and generates the 5-HETE series of metabolites, and that specific inhibitors of 5-Lox activity block production of metabolites and induces apoptosis. This apoptosis is prevented by exogenous 5(S)-HETE (5-hydroxyeicosatetraenoic acid), and more effectively by 5-oxoETE, a dehydrogenase-derivative of 5-HETE (25–28). Interestingly, inhibition of 5-Lox does not affect Akt or ERK, but rapidly inactivates PKCε, revealing a new fundamental mechanism of prostate cancer cell survival regulated by 5-Lox (29, 30). We also documented that the active 5-Lox metabolite (5-oxoETE) signals via a G protein-coupled oxoeicosanoid receptor (OXER1) followed by activation of protein kinase C-epsilon (30–32). We hypothesized that similar survival and anti-apoptosis mechanisms may also be operative in other types of cancer cells and are regulated by 5-Lox activity.

Since, the status and role of 5-Lox pathway and its downstream mechanisms in the pathobiology of pancreatic cancer is still unknown, systematic exploration of the involvement of, (1) the phosphatidylinositol 3’-kinase-Akt/protein kinase B (PI3K-Akt) pathway, and (2) the protein kinase C-epsilon (PKCε) pathway is needed. These two pathways are well-characterized to promote the growth and survival of a variety of cells, including cancer cells (33–40). The PI3K-Akt pathway is over-activated in many types of cancer cells and is well characterized to play an important role in the cellular signaling network regulating various functions including cell proliferation, growth, and metabolism. PI3K-Akt is well known to promote cell survival through defined apoptosis-preventing mechanisms (41–45). Similarly, the PKCε isoform is known to be oncogenic which promotes tumor growth and recurrence by increasing cell proliferation as well as by decreasing apoptosis. PKCε is a member of the PKC-family of serine-threonine protein kinases constituted by about ten isoforms which are known to regulate a variety of cell functions, such as cell proliferation, apoptosis, angiogenesis, carcinogenesis, metabolism, and cell motility (37–40). MK591 is a second-generation leukotriene biosynthesis inhibitor which inhibits the activity of 5-Lox via binding with the 5-Lox-activating protein (FLAP), thus blocking its ability to present the substrate, arachidonic acid, to 5-Lox for catalysis (46–50). Our previous findings suggest that MK591 inhibits the growth of prostate cancer cells via apoptosis (29, 32). MK591 is widely used because of its specificity to inhibit 5-Lox activity, and it does not show any detectable inhibition of the cyclooxygenase, epoxygenase or 12-lipoxygenase activities. Both lipoxygenase and cyclooxygenase play crucial roles in the synthesis of eicosanoids derived from arachidonic acid. To demonstrate the selective involvement of 5-LOX in pancreatic cancer cells, independent of COX-2, ibuprofen, (an inhibitor of cyclooxygenase), was utilized as a negative control which did not affect viability of cells or the production of 5-LOX. We were interested in testing MK591 as a targeted therapeutic agent for pancreatic cancer because it is orally bioavailable, and no significant off target effects of MK591 have been noted by molecular analysis (51).

These findings suggest that pancreatic cancer cells gain survival and growth advantages from the readily available arachidonic acid (abundant in high-fat “Western” diets) via generation of 5-Lox metabolites and that the survival-promoting effect of 5-Lox is exerted via downstream signaling involving the serine/threonine protein kinase, PKCε.

## 2 Materials and methods

### 2.1 Cell culture and reagents

BxPC3, Panc-1 and MiaPaCa-2 human pancreatic cancer cells were purchased from American Type Culture Collection (Manassas, VA). The KPC-1402 and KPC-1404 transgenic mouse-derived pancreatic cancer cell lines and KPC tumor slides were kindly provided by Dr. Mandar Muzumdar/Dr. Tyler Jacks (MIT, Cambridge, MA). These cancer cells were generated from pancreatic tumors isolated from KPC transgenic mice which are characterized by KrasG12D/+:Trp53R172H/+:Pdx-1-Cre (52). Cells were cultured in DMEM medium (Invitrogen, Carlsbad, CA) supplemented with 10% FBS and antibiotics. The 5-Lox metabolite, 5-OxoETE, was purchased from Cayman Chemicals (Ann Arbor, MI). Live lentiviral particles generating 5-Lox shRNA (small hairpin-RNA) were bought from Santa Cruz Biotech (Santa Cruz, CA). MK591 was generously provided by Dr. Robert N. Young (Merck-Frosst Centre for Therapeutic Research, Quebec, Canada) as a gift for *in vitro* and *in vivo* experiments.

### 2.2 Cell viability assay

Cells were plated overnight in 96 well plates (~5,000 cells per well) in complete growth medium (DMEM plus 10% FBS and antibiotics) and treated with varying doses of MK591 as indicated. Then, the plates were incubated further for 72 hours at 37°C in the CO2 incubator. Cell viability was measured by the MTS/PES One Solution Cell Titer assay from Promega (Madison, WI) as described previously (29–32). The half maximal inhibitory concentration (IC50) value of each cancer cell was determined from three independent determinations. To derive the IC50 values, a linear inhibition curve for MK591 was utilized.

### 2.3 Annexin-V binding

Cells (~3 x 10^5^) were plated in 60mm diameter tissue culture plates in RPMI medium and allowed to grow for 48 hours. Then the spent culture medium was replaced with fresh 2ml DMEM medium and the cells were treated with MK591 (40μM) or ibuprofen (40μM) for 24 hours at 37°C. Then the cells in the plate were treated with FITC-labeled annexin-V and propidium-iodide (PI) for 15 minutes in the dark using Annexin V-Binding Detection Kit following a protocol supplied by the manufacturer (BD Biosciences). After washing, cells were photographed with a Nikon digital camera attached to a LEICA fluorescence microscope at 20X. Images were acquired by a Nikon digital camera attached to a Zeiss fluorescence microscope and the data were processed with a Dell computer using Q-Capture-7 Pro software.

### 2.4 Western blot

Cells (~3 x 10^5^) were plated in 60mm diameter plates and allowed to grow for 48 hours. On the day of experiments, the old culture medium was replaced with 2ml fresh RPMI medium and then the cells were treated with MK591 or Ibuprofen (48 hours) or 5-Lox shRNAs (96 hours) as described in the respective experimental and results section. After the treatment period, cells were harvested, washed, and lysed in lysis buffer (50mM HEPES, pH 7.4, 150mM NaCl, 1mM EDTA, 1mM orthovanadate, 10mM sodium pyrophosphate, 10mM sodium fluoride, 1% NP-40, and a cocktail of protease inhibitors). Proteins were resolved by 12% SDS–PAGE and transferred to nitrocellulose membranes. Membranes were blocked with 5% nonfat-milk solution and then blotted with appropriate primary antibody followed by horseradish peroxidase-labeled secondary antibody. Bands were visualized by enhanced chemiluminescence detection kit from Pierce Biotech (Rockford, IL). Proteins of interest were analyzed by Western blot in three separate experiments (n=3), unless otherwise mentioned.

### 2.5 Measurement of PKCε activity by immunoprecipitation-kinase (IP-Kinase) assay

Pancreatic cancer cells (~1 x 10^6^) were plated in 100mm diameter plates and allowed to grow for 48 hours. On the day of experiment, the old culture medium was replaced with fresh 5ml RPMI medium and the cells were treated with MK591 (40μM) with or without 10μM 5-oxoETE for 6 h. Ibuprofen (40μM) was used in parallel as negative control. Then the cells were lysed in lysis buffer (50mM HEPES, pH 7.4, 150mM NaCl, 1mM EDTA, 1mM orthovanadate, 10mM sodium pyrophosphate, 10mM sodium fluoride) containing 0.4% Triton-X100, 10% glycerol, and a cocktail of protease inhibitors. Effect of MK591 on PKCε was analyzed by using pure PKCε treated with MK591 (40μM) and/or KIE1-1 (50μM), a specific peptide inhibitor of PKCε for 10 min at RT before measuring the kinase activity. Enzymatic activity of PKCε was measured by an ELISA method using biotin-labeled peptide substrate (Cell Signaling Technology, Danvers, MA) (30, 31). Briefly, cell lysates were cleared by centrifugation at 12,000 x g for 10 minutes at 4°C and the supernatants (~500μg proteins) were used for immunoprecipitation of PKCε using 4μg anti-PKCε antibody (Santa Cruz, CA). Tubes were gently rotated overnight at 4°C and the immunecomplexes were precipitated using anti-rabbit IgG-coated magnetic beads (Invitrogen) for two hours at 4°C. Then the beads were washed five times with lysis buffer containing 0.1% Triton-X100 and finally suspended in 25μl of 1X kinase assay buffer. Enzymatic reactions in 50μl were carried out for 15 minutes at room temperature (RT) using 10μl of IP-slurry with beads and stopped with 50μl of 50mM EDTA. Aliquots of reaction mixtures (25μl) were placed into streptavidin-coated 8-well strips and incubated for 60 minutes at RT. Wells were washed twice with PBS and phosphorylation of biotinylated-peptide substrate (cAMP response element-binding protein or CREB at Ser-133) was detected by specific anti-phosphoserine primary antibody followed by secondary HRP-labeled anti-rabbit antibody (Cell Signaling Technology, Danvers, MA). After washing, color was developed using ABTS (2,2’-azino-di (3-ethylbenzthiazoline-6-sulfonate) as substrate for 15 minutes at RT. Absorbance was measured at 405nm in a digital microplate reader (Bio-Tek Instruments).

### 2.6 Invasion assay

*In vitro* invasion of Panc-1 cells was measured using modified Boyden chambers following methods we published previously (29–32). Briefly, chambers were soaked in 50µl serum-free DMEM medium and 40,000 cells were placed in the upper chamber. The chambers were then placed on top of the lower chamber, containing 500ul of DMEM medium containing 2% FBS as attractant. The assembly with untreated and MK591-treated cells (20 or 40µM) were incubated in the CO2 incubator for 24 hours. At the end of incubation, the un-invaded cells along with Matrigel in the upper chambers were scrapped out and the membranes with invaded cells were fixed in methanol and stained with crystal violet. Then the membranes were cut and mounted on glass slides and pictures were taken with a Leica inverted microscope. Total invaded cells were counted in each membrane for quantitative measurement of treatment effects.

### 2.7 Measurement of apoptotic DNA degradation

Apoptosis was quantitatively measured by detecting degradation of chromatin-DNA to nucleosomal fragments using an assay kit based on sandwich-ELISA. Panc-1 cells (~3 x 10^5^) were plated in 60mm tissue culture plates and allowed to grow for 48 hours. Then, the cells were treated with the MK 591 (10-40µM) or solvent vehicle (DMSO) for varying periods of time up to 24 hours. At the end of incubation periods, cells were lysed in lysis buffer provided by the manufacturer and the degradation of chromatin-DNA to nucleosomal fragments was measured by Cell Death Detection ELISAplus kit from Roche (Indianapolis, IN) as described before (29–32).

### 2.8 *In vivo* tumor models

To analyze the effect of MK591 on pancreatic tumor growth, exponentially growing Panc-1cells or luciferase labeled MiaPaCa2 cells (MiaPaCa2-Luc) were injected subcutaneously into the flanks of 8 weeks old Balb/c nude mice (n=6). When the tumors grew to approximately 100 mm^3^, mice were randomized and treated with vehicle (1:1:8 solution of DMSO : Cremophor:PBS) or MK591 (200 mg/kg/day) for 4 weeks via oral gavage. Tumor growth was monitored by measuring volumes using a digital slide-calipers. Tumor measurements were taken once every week and volumes were calculated by the formula v = L x (W)^2^/2. The luciferase activities in the tumors were measured by intra-peritoneal injection of 100 microliter of a 3mg/ml luciferin solution followed by imaging in a Kodak Care-Stream imaging station. X-ray images were also taken keeping animals in the same supine position. Representative pictures of a control and treated mice are shown here.

### 2.9 Immunohistochemistry

Slides with sections fixed in formalin and embedded in paraffin were heated to 60°C for one hour. Antigen retrieval was then performed using EnVision FLEX target retrieval solution with low pH (Agilent Dako, S236984–2) in a PT Link device (Agilent Dako, PT200). The slides were subsequently rinsed in 1X TBST wash buffer for 5 minutes, followed by a 5-minute application of Peroxidazed 1 (Biocare Medical, PX968 M) to quench endogenous peroxidase activity. To block non-specific background staining, slides were treated with Background Punisher (Biocare Medical, BP974) for 10 minutes. The primary antibodies targeting specific proteins (5-LOX antibody, CST#3289; p-MAPK (Erk1/2) antibody, CST#9101; Aurora A antibody, CST#91590; Cyclin D1 antibody, Santa Cruz Biotechnology sc-8396; Ki67 antibody, Vector Laboratories VP-RM04; PKC epsilon antibody, Santa Cruz Biotechnology sc-1681) were diluted in EnVision FLEX Antibody Diluent (Agilent Dako, K800621–2; IgG Isotype Control CST#3900) and incubated overnight at 4°C. After washing, slides were incubated with Mach2 Double Stain 1 or 2 (Biocare Medical, MRCT523/525) for 30 minutes at room temperature. Development of the slides was achieved using ImmPACT DAB Substrate, Peroxidase (HRP) (Vector Labs, SK-4105), followed by a 5-minute counterstaining with Hematoxylin (Agilent DAKO, K800821–2). Finally, the slides were washed with distilled water, dried, and mounted using EcoMount (Biocare Medical, EM897 L).

### 2.10 Drug synergy analysis

The cells were co-treated with specified doses of MK591 and gemcitabine, and the cytotoxic effects of both the individual agents and their combinations were assessed via MTS assay. The synergy of the drugs was evaluated using the SynergyFinder 2.0 software (53). Interactions between the drugs are categorized based on their synergy score: scores above +10 indicate synergistic interactions, scores from -10 to +10 suggest additive effects, and scores below -10 denote antagonistic interactions. Deviations between the expected and observed responses, marked by positive δ-values (red areas) and negative δ-values (green areas), signify synergy and antagonism, respectively.

### 2.11 Statistical analysis

Results are presented as mean values of each data point ± SEM (standard error of the mean). The statistical significance of the difference between different groups were calculated by One-way ANOVA followed by Tukey’s multiple comparison test using GraphPad Prism8.0 software. A p value of <0.05 was considered as statistically significant.

## 3 Results

### 3.1 Pancreatic cancer cells and tumors expressed high levels of 5-Lox and inhibition of 5-Lox decreased the viability of pancreatic cancer cells

**Figure 1:**
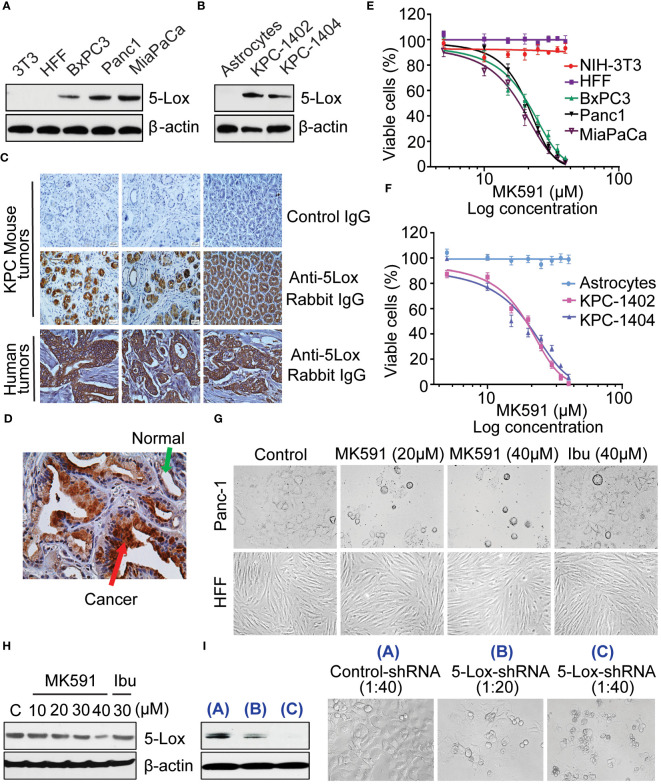
Effect of MK591 on the viability of pancreatic cancer cells. **(A, B)** Protein level of 5-Lox in different cell lines was analyzed by Western blot. **(C, D)** Expression of 5-Lox protein in KPC mouse and human pancreatic tumors was detected by IHC. The images were taken at 300x magnification (Objective lens (30x) × eyepiece (10x)). **(E, F)** The IC50 values for MK591 were found to be 21.89 µM for BxPC3, 20.28 µM for Panc-1, and 18.24 µM for MiaPaCa-2 human pancreatic cancer cell lines, respectively. Against KPC-1402 and KPC-1404, the IC50 values of MK591 were determined to be 19.02 µM and 20.41 µM, respectively. The IC50 values for MK591 against normal cells such as NIH-3T3 and HFF were found to be 768.57 µM and 1524.92 µM, respectively. **(G)** Phase contrast morphology of cancer (Panc-1) and normal (HFF) cells with and without MK591 treatment. Cells were treated with MK591 for 72 hours at 37°C in the CO2 incubator. **(H)** Protein level of 5-Lox in MK591 treated (48 hours) Panc-1 cells was analyzed by Western blot. **(I)** The effect of MK591 on 5-Lox protein was detected by Western blot. Results are shown as mean values of each data point ± standard error (n = 3).

Analysis of human pancreatic cancer cell lines by western blot revealed that these cells express high amount of 5-Lox protein, however the expression level of 5-Lox proteins in normal fibroblasts was undetectable (**Figure 1A**). Moreover, transgenic mouse pancreatic tumor-derived cells (KP-1402 and KP-1404) showed high levels of 5-Lox protein (**Figure 1B**). Using a mAb for 5-Lox, the immunohistochemical analysis showed that both mouse and human pancreatic tumors express high levels of 5-Lox with an intense positive stating in ductal and islet cells (**Figure 1C**). No significant staining was detected in healthy pancreatic ductal cells or negative controls. (**Figure 1D**). Interestingly, MK591 (46–51), decreases the viability of pancreatic cancer cells in a dose-dependent manner (**Figures 1E-G**). The IC50 values for MK591 across different pancreatic cancer cell lines are detailed in the legend of **Figure 1**. The IC50 values for MK591 were found to be 21.89 µM for BxPC3, 20.28 µM for Panc-1, and 18.24 µM for MiaPaCa-2 human pancreatic cancer cell lines, respectively. Against KPC-1402 and KPC-1404, the IC50 values of MK591 were determined to be 19.02 µM and 20.41 µM, respectively. In contrast, MK591 does not affect normal/non-cancer cells, which do not express detectable 5-Lox proteins under normal culture conditions, suggesting that the expression and function of 5-Lox is cancer-specific. The IC50 values for MK591 against normal cells such as NIH-3T3 and HFF were found to be 768.57 µM and 1524.92 µM, respectively. Treating pancreatic cancer cells with either 20 or 30μM MK591 resulted in only a slight decrease in 5-Lox protein levels, as determined through Western blot analysis (**Figure 1H**), however this treatment had a strong effect on inducing cell (**Figures 1E, F**). A decrease in the viability of pancreatic cancer cells was also observed when the expression level of 5-Lox was decreased by treatment with lentiviral 5-Lox-targeting shRNA (**Figure 1I**). Similar results were observed with two other pancreatic cancer cell lines, BxPC3 and MiaPaCa-2 (data not shown).

### 3.2 Inhibition of 5-Lox induced apoptosis in pancreatic cancer cells

**Figure 2:**
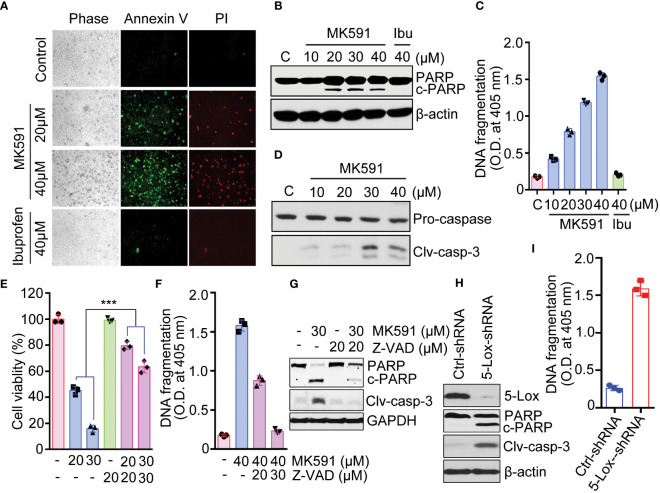
Induction of apoptosis in pancreatic cancer cells by MK591. **(A)** Panc-1 cells were treated either with MK591 (40μM) or ibuprofen (40μM) at 37°C for 48 hours. Control cells were treated with vehicle only (0.2% DMSO) (200× magnification). **(B)** Cells were treated with indicated doses of MK591 for 48 hours. **(C)** Degradation of chromatin DNA to nucleosomal fragments was detected by ELISA. **(D)** Cleaved caspase-3 was detected in Panc-1 cells treated with MK591 for 48 hours. **(E-G)** MK591 induced Panc-1 cell viability **(E)**, apoptosis **(F)**, and cleavage of PARP and Caspase 3 proteins **(G)** were inhibited by Z-VAD-FMK, a pan-caspase inhibitor. **(H)** Panc-1 cells were treated with control or 5-Lox shRNA lentiviral particles. After 96 hours, cleaved PARP protein was detected by Western blot. **(I)** Panc-1 cells were treated as in **(H)** and effect of 5-Lox knockdown on apoptotic DNA fragmentation was measured using an ELISA kit (Roche). Results are shown as mean values of each data point ± standard error (n = 3). ***p<0.0005, determined by One-way ANOVA followed by Tukey’s multiple comparison test.

A distinct positive binding of annexin-V with Panc-1 cells was observed after treatment with MK591 (**Figure 2A**). The characteristic cleavage of PARP (poly-ADP ribose polymerase) proteins occurred in pancreatic cancer cells treated with doses of MK591 (**Figure 2B**). Further analysis of MK591 treated pancreatic cancer cells, revealed degradation of nuclear chromatin DNA to oligo-nucleosomes (**Figure 2C**). In similar experimental conditions, cells treated with ibuprofen did not show any signs of apoptotic cell death (**Figures 2A-C**). These findings suggest that the effect of 5-Lox inhibition to induce apoptosis in pancreatic cancer cells is selective. Furthermore, MK591 induced proteolytic cleavage of caspase 3 in pancreatic cancer cells and that MK591 induced apoptosis is blocked when the cells were treated with the caspase inhibitor, Z-VAD-FMK (**Figures 2D-G**). Intriguingly, inhibition of 5-Lox using shRNA also resulted in cleavage of PARP (**Figure 2H**) and degradation of nuclear-DNA to nucleosomal fragments (**Figure 2I**). Taken together, these experiments indicate that pharmacological or genetic inhibition of 5-Lox induces apoptotic cell death in pancreatic cancer cells.

### 3.3 Inhibition of 5-Lox inhibited *in vitro* invasion and soft-agar colony formation by pancreatic cancer cells

**Figure 3:**
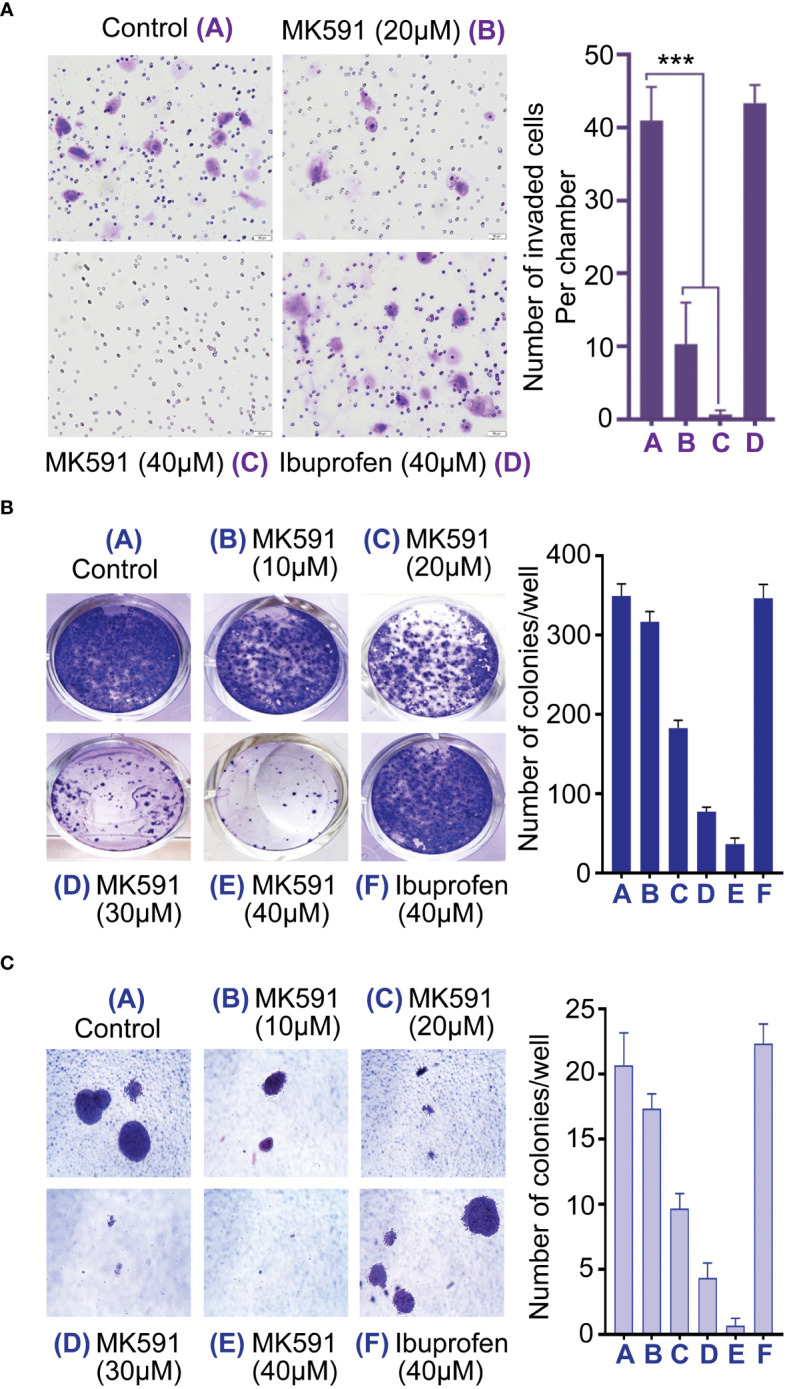
Effect of MK591 treatment on invasion and soft-agar colony formation. **(A)** Effects of MK591 on *in vitro* invasion of Panc-1 cells. Invaded cells were analyzed after staining with 0.025% crystal violet. **(B)** Colony growth of MK591 treated Panc-1 cells. **(C)** Effect of MK591 on soft-agar colony formation of Pacn-1 cells. Results represent mean values of each data point ± standard error (n = 3). ***p<0.0005, determined by One-way ANOVA followed by Tukey’s multiple comparison test.

The study observed that sub-lethal doses of MK591 dramatically impaired the invasive capabilities of pancreatic cancer cells *in vitro*, as depicted in **Figure 3A**. This finding was further corroborated by the remarkable reduction in the development of cell clusters, known as foci, on the surfaces of cell culture plates (**Figure 3B**). Additionally, the ability of these cancer cells to form colonies within a soft-agar medium was also dramatically inhibited by MK591 in a dose-dependent manner (**Figure 3C**).

### 3.4 MK591 inhibited pancreatic tumor growth in nude mice xenografts

**Figure 4:**
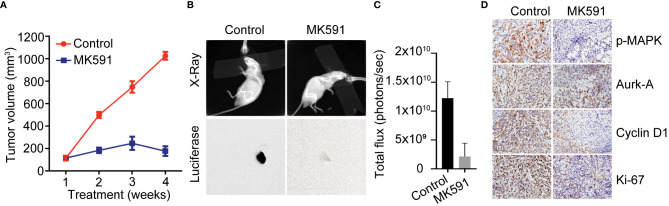
*In vivo* effects of MK591 on pancreatic tumor growth in xenografts. **(A)** Tumor measurements were taken once every week and tumor volumes were calculated. **(B)** Luciferase activities in the tumors were measured by imaging in a Kodak Care-Stream imaging station. X-ray images were also taken keeping animals in the same supine position. **(C)** Bioluminescence intensities from xenograft tumors at the end of the experiment. **(D)** Expression levels of p-ERK/MAPK, Aurora kinase, cyclin D1 and Ki-67 were detected by IHC. Data are represented as mean ± standard deviation (n = 6).

MK591 dramatically inhibited tumors growth developed by Panc-1 and MiaPaCa-2 cells in mouse xenografts when given at a dose of 200mg/kg/day via oral gavage for four weeks (**Figures 4A, B**). Moreover, a strong inhibition in the levels of p-ERK, Aurora kinase-A, and cyclin D1 was observed in MK591 treated tumors, compared to normal controls (**Figure 4C**). Additionally, analysis of tumor tissues for the cell proliferation marker showed a significant decrease in Ki-67 positive cells in MK591-treated mice compared to control mice (**Figure 4D**).

### 3.5 Inhibition of 5-Lox downregulated PKCε and K-Ras, but did not inhibit Akt in pancreatic cancer cells

**Figure 5:**
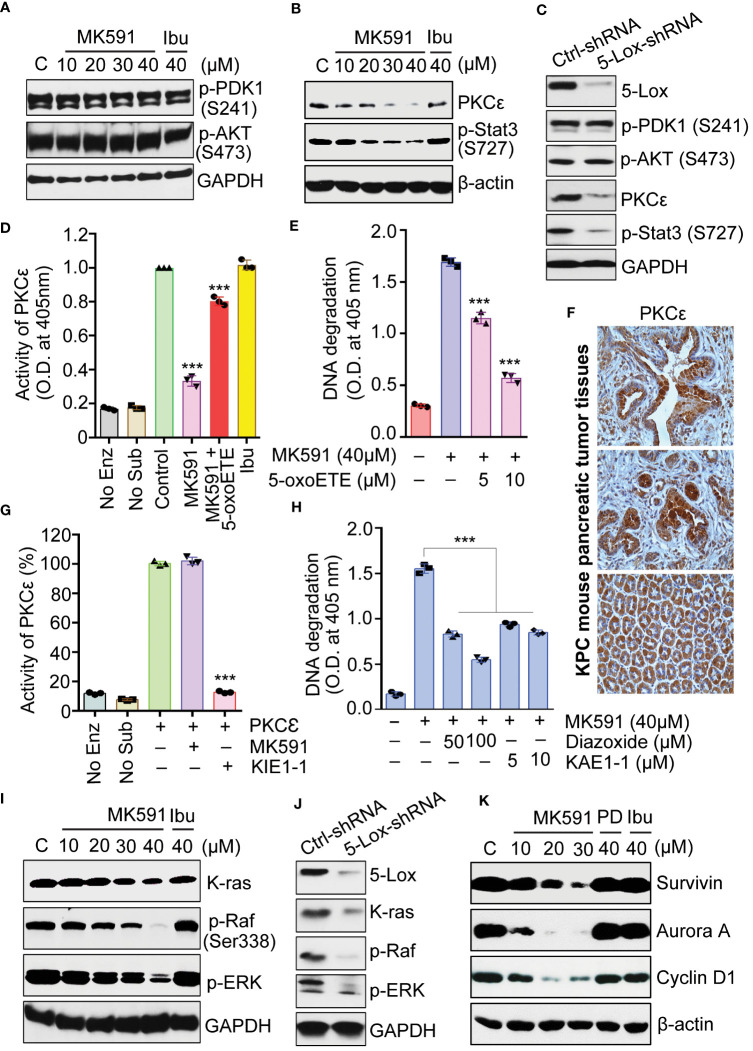
Effects of MK591 on Akt, PKCε and the K-Ras signaling pathway in pancreatic cancer cells. **(A, B)** Panc-1 cells were treated as indicated for 48 hours, and the control cells were treated with vehicle only (0.2% DMSO) for protein analysis. **(C)** Panc-1 cells treated with control or 5-Lox shRNA lentiviral particles for 96 hours. **(D)** Panc-1 cells were treated with vehicle only (0.2% DMSO), MK591 (40μM) with or without 10μM 5-oxoETE for 6 hours. Ibuprofen (40μM) was used as negative control. **(E)** Panc-1 cells were treated with or without the addition of 5-oxoETE for 24 hours and apoptosis was measured by ELISA. **(F)** Representative photographs from different animals showing the expression levels of PKC-epsilon protein by IHC. **(G)** Effect of MK591 on PKCε was analyzed by using pure PKCε treated with MK591 (40μM) and/or KIE1-1 (50μM). **(H)** Panc-1 cells were treated as indicated for 24h. Apoptosis was measured by Cell Death ELISA. **(I)** Panc-1 were treated as indicated for 48 hours. Control cells were treated with vehicle only (0.2% DMSO). **(J)** Panc-1 cells were treated with control or 5-Lox shRNA lentiviral particles for 96 hours and analyzed for indicated proteins by western blot. **(K)** Panc-1 cells were treated as indicated in **(A)** and the protein levels were detected by western blot. Data presented as mean values ± standard error (n=3). ***p<0.0005, determined by One-way ANOVA followed by Tukey’s multiple comparison test.

Interestingly, neither the phosphorylation of Akt nor its upstream kinase PDK1, was inhibited when cells undergo apoptosis by treatment with MK591 or by 5-Lox shRNA (**Figures 5A, C**). This finding provoked us to examine the involvement of PKCε, which was reported to promote survival and to decrease apoptosis in a variety of cell types (37–40). To investigate the potential role of PKCε as a downstream mediator of the survival-promoting effects of 5-Lox, pancreatic cancer cells were treated with MK591 or 5-Lox shRNA which showed a decrease in the expression of PKCε (**Figures 5B, C**). Moreover, these cells showed a rapid decrease in the phosphorylation of Stat3 at Serine-727, a site phosphorylated by PKCε, suggesting that 5-Lox activity may regulate PKCε in pancreatic cancer cells. This regulation was further confirmed by using metabolic products of 5-Lox which are expected to reverse the inhibitory effects of MK591 in pancreatic cancer cells. Therefore, pancreatic cancer cells were treated with 5-oxoETE, a metabolic product of 5-Lox activity and then treated with MK591. Subsequently, 5-oxoETE efficiently prevented MK591-induced inhibition of PKCε activity as well as the induction of apoptosis in pancreatic cancer cells (**Figures 5D, E**), suggesting a critical role for 5-Lox activity in the regulation of these processes. Furthermore, the pronounced expression of PKCε protein in pancreatic tumors of transgenic KPC mice corroborated the potential involvement of the 5-Lox > PKCε pathway in pancreatic cancer progression (**Figure 5F**).

An interesting observation about MK591 was that it does not affect the enzymatic activity of PKCε when directly added to the assay mixture containing immunoprecipitated PKCε from Panc-1 cells, suggesting that MK591 does not inhibit the activity of pure PKCε through off-target effect (**Figure 5G**). Moreover, when pancreatic cancer cells were pre-treated with diazoxide (a chemical activator of PKCε) or KAE1-1 (a peptide activator of PKCε), the 5-Lox inhibition-induced apoptosis was effectively prevented (**Figure 5H**). The effect of 5-Lox inhibition on the K-Ras pathway was examined and found that 5-Lox inhibition by MK591 or shRNA dramatically inhibits the protein level of K-Ras and downstream activating phosphorylation of c-Raf and ERKs in pancreatic cancer cells (**Figures 5I, J**). Moreover, the expression of downstream molecular targets of the K-Ras pathway, including survivin, aurora A kinase, and cyclin D1, was found to diminish following MK591 treatment (**Figure 5K**).

### 3.6 Inhibition of 5-Lox synergized with gemcitabine to induce apoptosis in pancreatic cancer cells

**Figure 6:**
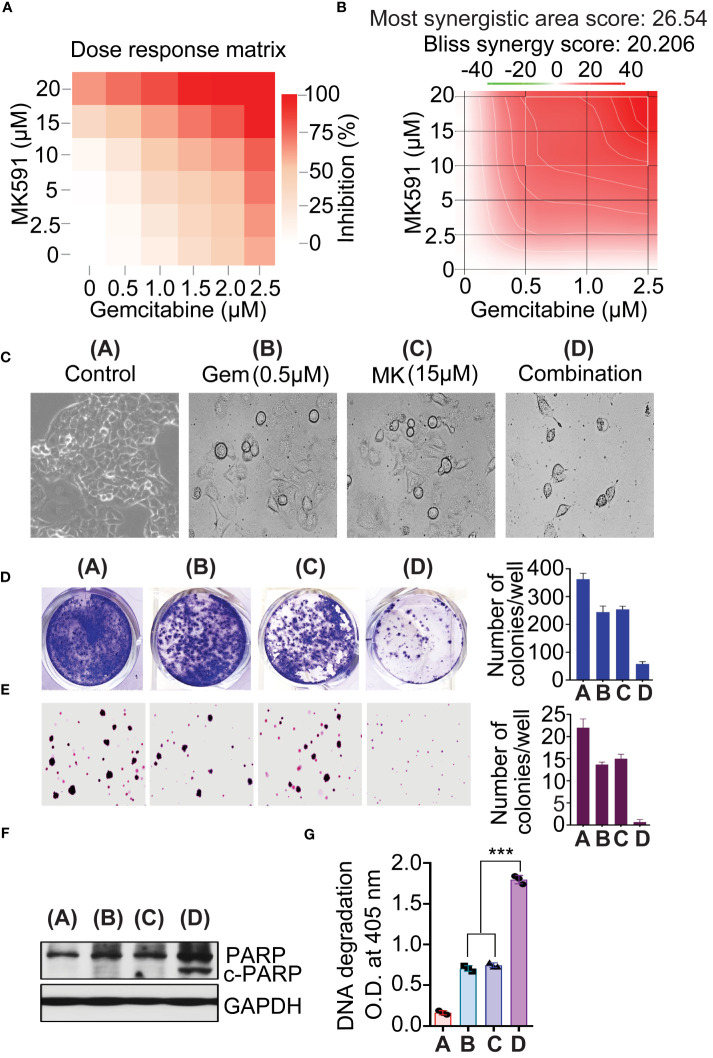
MK591 enhances sensitivity to gemcitabine in pancreatic cancer cells. **(A, B)** Panc-1 cells were treated as indicated for 72 hours. The 3D synergy landscape generated by bliss model. Cell viability was measured by MTS/PES assay. Note: Red surfaces denote a synergistic interaction, and green surfaces an antagonistic interaction. **(C)** Phase contrast morphology of Panc-1 cells after treatment with single agents or their combination as indicated. **(D)** Colonies were stained with 0.025% crystal violet. **(E)** Soft-agar colony formation of Pacn-1 cells was measured with indicated doses for 21 days. **(F)** Panc-1 cells were treated as indicated for 48 hours. **(G)** Degradation of chromatin DNA to nucleosomal fragments was detected by ELISA. Results represent mean values of each data point ± standard error (n = 3). ***p<0.0005, determined by One-way ANOVA followed by Tukey’s multiple comparison test.

The combination of MK591 and gemcitabine was found to significantly reduce the survival of pancreatic cancer cells compared to the use of each agent alone, as shown by the Bliss synergy model (**Figures 6A-C**). To determine whether 5-Lox inhibition and gemcitabine attenuate the ability of pancreatic cancer cells, the foci and soft agar colony-forming abilities of Panc-1 cells treating with MK591 and gemcitabine individually and in combination were examined. Co-treatment of MK591 and gemcitabine drastically reduced colony formation (**Figures 6C-E**) and increased apoptosis over single-agent treatments in pancreatic cancer cells, as seen by characteristic cleavage of PARP and DNA fragmentation (**Figures 6F, G**).

## 4 Discussion

It is interesting to note that under healthy conditions, the expression and transient activation of 5-Lox are usually confined to certain immune cells, including neutrophils, basophils, eosinophils, and macrophages. In these cells, another category of 5-Lox metabolites, namely leukotrienes, are pivotal in facilitating chemotaxis. In contrast, the vast majority of non-immune cells, known as parenchyma cells, throughout the body do not show any noticeable 5-Lox activity under normal situations. The expression of 5-Lox in these cells only becomes apparent during inflammatory conditions such as asthma, arthritis, or cancer (54–58). In this light, this study reveals a pronounced increase in the expression and activity of 5-Lox in pancreatic cancer tissues compared to the adjacent non-cancerous tissues. This observation, coupled with the critical role of 5-Lox in the survival of pancreatic cancer cells, indicates that 5-Lox may play a significant role in the development and progression of pancreatic cancer. Consequently, targeting 5-Lox presents a promising strategy for the effective treatment of advanced and deadly pancreatic cancer. This finding aligns with the broader observation that, although 5-Lox is typically not expressed in the non-immune parenchymal cells of healthy individuals, its overexpression is linked to various inflammatory conditions and cancers, such as asthma, arthritis, psoriasis, prostate cancer, lung cancer, pancreatic cancer, and glioma (19, 24, 54–58).

A dramatic reduction in the viability of pancreatic cancer cells was observed when the cells were treated with MK591, or by 5-Lox-specific shRNA, which demonstrates that pancreatic cancer cells depend on 5-Lox activity for their survival. Blocking 5-Lox triggered apoptosis in these cells, evidenced by PARP protein cleavage, phosphatidylserine externalization, and chromatin-DNA degradation into mono-/oligo-nucleosomes, which are considered as a hallmark of apoptotic cell death. In this study, MK591 was shown to effectively kills pancreatic cancer cells via apoptosis while sparing normal cells such as NIH3T3 fibroblasts, astrocytes, and human foreskin fibroblasts (HFF), demonstrating its cancer-specific action. Thus, through this work, MK591 has emerged as a new, targeted agent for pancreatic cancer therapy. Pancreatic cancer cells are highly metastatic which involves coordinated actions of cancer cell motility as well as degradation of extracellular matrix proteins. The capacity for invasion through extracellular matrix and anchorage-independent colony formation on soft-agar are characteristics of metastatic cancer cells. It was observed that MK591 dramatically decreased the invasive capability of pancreatic cancer cells at sub-lethal doses in a clear dose-dependent manner. Moreover, MK591 strongly inhibits the clonogenic and soft-agar colony forming abilities of pancreatic cancer cells. Furthermore, MK591 effectively reduced clonogenic growth and inhibited tumor growth in pancreatic cancer xenograft models without negatively impacting the animals’ overall health. Molecular analysis of residual tumors treated with MK591 revealed reduced levels of ERK, cyclin D1 and Aurora kinase B proteins, suggesting that targeting 5-Lox by MK591 or similar agents could be a potent strategy for inhibiting pancreatic tumor growth.

Though a critical role for 5-Lox in the survival of pancreatic cancer cells is now well understood, how 5-Lox provides survival signaling to these cancer cells is yet to be thoroughly elucidated. Signaling via the PI3K-Akt axis is well characterized as a pro-survival mechanism which plays an important role in the viability of a range of cell types, including normal as well as cancer cells (41–45). Since, Akt has emerged as a valid target, a series of compounds have been explored or developed for both mechanistic study and clinical trials aimed at cancer treatment. However, this research revealed that blocking 5-Lox, either through pharmacological means or genetic modification, does not affect the phosphorylation of Akt (at Ser-473) or its upstream kinase PDK1 (at Ser-241), despite inducing apoptosis**.** The findings suggest that the induction of apoptosis in pancreatic cancer cells by inhibiting 5-Lox occurs independently of Akt activity suppression. This novel finding not only indicated that an additional mechanism exists in pancreatic cancer cells to mediate the effects of 5-Lox, but also it suggested that the 5-Lox activity feeds a survival mechanism which may help pancreatic cancer cells to bypass chemotherapies that are directed against Akt. However, details about the identity and characteristics of a possible kinase behind this Akt-independent mechanism of pancreatic cancer cell survival were unknown. These observations piqued our interest in exploring further mechanisms underlying the induction of apoptosis in pancreatic cancer cells by 5-Lox inhibition and invoked us to examine the role of PKCε as a potential mediator. This research delved into the role of PKCε in pancreatic cancer cell survival, prompted by its known function in enhancing survival and growth in various cell types, including cancer cells A marked reduction in PKCε protein levels was noted when pancreatic cancer cells are treated with MK591 or 5-Lox shRNA. This observation was further supported by a decrease in the phosphorylation of Stat3 at serine-727, a known target of PKCε (36–40). Interestingly, both the inhibition of PKCε activity and the induction of apoptosis by MK591 treatment, are inhibited by exogenous 5-Lox metabolites, suggesting that 5-Lox activity regulates survival of pancreatic cancer cells via PKCε. These experiments also demonstrated that MK591 does not inhibit the activity of isolated PKCε enzyme by direct interactions, which ruled out the possibility of any off-target effect of MK591 on PKCε enzymatic activity. The application of both a chemical (diazoxide) and a peptide (KAE1-1) activator of PKCε effectively averted apoptosis caused by 5-Lox inhibition, reinforcing the idea that 5-Lox’s role in supporting pancreatic cancer cell survival operates through a PKCε-dependent pathway. Further investigations revealed that inhibiting 5-Lox through MK591 or shRNA significantly reduced the activation of key components in the K-Ras signaling pathway, notably c-Raf and ERKs, which are critical in pancreatic cancer progression. Given the pivotal role of the K-Ras pathway in pancreatic cancer and the complexities associated with directly targeting K-Ras due to its non-enzymatic nature, these findings propose a novel approach to disrupting this pathway through targeting 5-Lox with specific agents like MK591.

The PKCε isotype is well characterized as a transforming oncogene and it promotes cell survival through the regulation of Bcl-2 family members such as Bcl-2, Bad, and Bax to increase resistance to apoptosis-inducing agents (34, 35, 59–63). A recent gene knockdown study revealed that PKCε is an important regulator for pancreatic cancer lesion development in transgenic mice (64). The remarkable cancer-selective expression in pancreatic epithelial cells, together with a critical role of 5-Lox activity in the survival of pancreatic cancer cells, suggest for a pivotal and possibly an indispensable role of 5-Lox in the development and progression of pancreatic cancer. Thus, 5-Lox has emerged as a novel, promising target for pancreatic cancer therapy. Given the prevalence of arachidonic acid, an omega-6 polyunsaturated fatty acid, in Western diets—where pancreatic cancer is also notably common—and the overexpression of 5-Lox in pancreatic cancer cells, these findings implicate the metabolism of arachidonic acid through the 5-Lox pathway as a significant contributor to pancreatic cancer’s pathobiology by enhancing the protean effects of PKCε. These studies have shown that inhibiting PKCε in pancreatic cancer cells with the 5-Lox inhibitor MK591—and the subsequent reversal of this inhibition by 5-oxoETE and the prevention of MK591-induced apoptosis through chemical and peptide activators of PKCε—demonstrate that apoptosis induced by 5-Lox inhibition in these cells occurs via the inhibition of PKCε. Given PKCε’s pleiotropic effects of PKCε on cancer cell survival and growth, this data further suggests that the survival- and growth-promoting effects of arachidonate 5-Lox in pancreatic cancer cells are mediated through PKCε and its downstream signaling pathways. Thus, a fundamental mechanism of pancreatic cancer cell survival has been unveiled, and since the activity of PKCε in pancreatic cancer cells is regulated by inhibitors and metabolites of 5-Lox, these findings opened a new research avenue for understanding the activation mechanism of PKCε in pancreatic cancer for therapeutic intervention.

From this work it is apparent that a characteristic feature of pancreatic cancer cells is the maintenance of a continuously active form of PKCε, as well as the phosphorylation/activation of its kinase substrate, Stat3. This phenomenon has also been observed in prostate cancer cells, where PKCε demonstrates membrane localization and enzymatic activity (30, 31, 65). The mechanism by which PKCε remains perpetually active in pancreatic cancer cells poses an intriguing question. According to the generally accepted paradigm, the activation mechanism of PKCε involves two sequentially aligned steps. In the first step, the PKCε protein is phosphorylated at a threonine residue at the activation loop by the phospholipid-dependent kinase1 (PDK1). This phosphorylation leaves PKCε in an inactive state in the cytosol, as its active site is blocked by a pseudo-substrate region. Activation is achieved when PKCε binds to a lipid second messenger like diacylglycerol (DAG) in a membrane environment, enabling it to phosphorylate its substrates. Upstream signaling mechanisms that may feed PKCε for its continuous activity in pancreatic cancer cells are yet to be fully characterized. However, production of 5-Lox metabolites from arachidonic acid and activation of OXER1, a G protein-coupled receptor (GPCR), for which the 5-Lox metabolites 5-oxoETE and to a lesser extent 5(S)-HETE serve as ligands, may ideally can provide the signals needed for the continuous activation of PKCε (31). Based on these correlative findings, we hypothesize that 5-Lox metabolites support the survival of pancreatic cancer cells through GPCR, OXER1 signaling and subsequent diacylglycerol (DAG) production for PKCε activation. Nonetheless, further experiments are needed to substantiate the validity of this hypothesis. Gemcitabine is frequently prescribed as a standard treatment of pancreatic cancer which works via induction of DNA damage/fragmentation in pancreatic cancer cells. Despite being the initial treatment choice, its efficacy is limited by rapid development of resistance that attributes to the evasion of apoptosis and eventually the disease assumes a lethal phenotype. Based on the observation that inhibiting 5-Lox can kill pancreatic cancer cells through apoptosis and block K-Ras signaling pathways, it was hypothesized that 5-Lox inhibition combined with gemcitabine could synergize to induce apoptosis and reduce the oncogenic phenotypes in pancreatic cancer cells. This research indicates that MK591, when used in combination with gemcitabine, enhances the induction of apoptosis in pancreatic cancer cells without overt toxicity to other non-cancer cells in the body, suggesting that a suitable combination of these two drugs at lower doses may improve gemcitabine’s therapeutic impact by overcoming the resistance mechanisms in pancreatic cancer cells.

In conclusion, inhibiting the 5-Lox pathway offers notable therapeutic promise for treating pancreatic cancer by triggering apoptosis. This process disrupts the survival and proliferation of cancer cells through the downregulation of K-Ras and subsequent inhibition of c-Raf and ERK phosphorylation, highlighting the 5-Lox pathway’s crucial role. The study reveals that 5-Lox inhibition blocks a PKCε-dependent survival mechanism, independent of Akt, emphasizing the significance of the 5-Lox pathway in controlling pancreatic cancer cell survival through PKCε signaling. Additionally, the enhanced efficacy of combining MK591 with gemcitabine suggests an effective combination therapy. These insights advocate for targeting the 5-Lox activity with specific inhibitors (such as MK591, AM679) might offer a promising and strategic approach in pancreatic cancer treatment, opening new avenues for therapy development.

## Data availability statement

The raw data supporting the conclusions of this article will be made available by the authors, without undue reservation.

## Ethics statement

Ethical approval was not required for the studies on humans in accordance with the local legislation and institutional requirements because only commercially available established cell lines were used. The animal study was approved by the institutional IACUC committee. The study was conducted in accordance with the local legislation and institutional requirements.

## Author contributions

JM: Conceptualization, Data curation, Formal analysis, Investigation, Methodology, Software, Validation, Visualization, Writing – original draft, Writing – review & editing. RGh: Data curation, Formal analysis, Investigation, Methodology, Validation, Visualization, Writing – review & editing. RGu: Data curation, Formal analysis, Investigation, Methodology, Validation, Visualization, Software, Writing – review & editing. DC: Data curation, Formal analysis, Investigation, Methodology, Validation, Visualization, Writing – review & editing. GK: Data curation, Formal analysis, Investigation, Methodology, Validation, Visualization, Writing – review & editing. JG: Data curation, Formal analysis, Investigation, Methodology, Validation, Visualization, Conceptualization, Funding acquisition, Project administration, Resources, Software, Supervision, Writing – original draft, Writing – review & editing.
